# Evaluation of the efficacy of ^18^F-FDG PET-CT combined with CT in detecting lymph node metastasis in liver cancer

**DOI:** 10.3389/fonc.2025.1636566

**Published:** 2025-12-18

**Authors:** Jun Yu, Xing-guo Tan, Fang Li

**Affiliations:** 1 Department of Hepatobiliary Surgery, Yueyang Affiliated Hospital of Hunan Normal University, Yueyang, China; 2 Department of Imaging, Yueyang Affiliated Hospital of Hunan Normal University, Yueyang, China

**Keywords:** diagnosis, efficacy, liver cancer, lymph node metastasis, PET-CT

## Abstract

**Background:**

This study aims to evaluate the diagnostic efficacy of ^18^F-FDG PET-CT imaging and enhanced abdominal CT scans for the preoperative detection of lymph node metastasis in liver cancer.

**Methods:**

We sought to compare the diagnostic performance of 18F-FDG PET-CT with that of CT and to determine the optimal predictive thresholds for lymph node metastasis, based on the maximum standardized uptake value (SUVmax) and the nodal short-axis diameter.

**Results:**

The diagnostic efficacy of ^18^F-FDG PET-CT, including sensitivity, specificity, and accuracy, was significantly higher than that of CT, with statistically significant differences (*P* < 0.05). Both the short diameter of lymph nodes and the SUVmax in the lymph node metastasis group were both greater than those in the non-metastasis group, with statistically significant differences (*P* < 0.05). The CT parameter of lymph node short diameter and the 18F-FDG PET-CT parameter of SUVmax were identified as independent predictors of lymph node metastasis in liver cancer and demonstrated a significant positive correlation (*P* < 0.001). The area under the receiver operating characteristic curve (ROC) for combined detection was 0.938, with a sensitivity of 92.3%, specificity of 85.3%, and accuracy of 88.3% for diagnosing regional lymph node metastasis in liver cancer. The efficacy of combined detection for diagnosing regional lymph node metastasis in liver cancer was superior to that of individual tests (*P* < 0.05), providing valuable clinical guidance for staging, treatment, and prognosis of liver cancer.

**Conclusion:**

The application of the optimal threshold values can further enhance the diagnostic accuracy of ^18^F-FDG PET-CT in detecting regional lymph node metastasis. The proposed criteria for lymph node metastasis were an SUVmax greater than 2.25 or a short diameter exceeding 8.5 mm. This information may assist in the formulation and optimisation of treatment plans for patients with liver cancer.

## 1 Introduction

Liver cancer is the sixth most common malignant tumour worldwide and the fourth leading cause of cancer-related mortality (1). Common risk factors include viral infections, such as hepatitis B and C viruses (HBV and HCV), chronic liver diseases (CLD) such as cirrhosis, alcoholism, and metabolic disorders including diabetes (2). Early-stage liver cancer often presents with subtle symptoms, and by the time it is diagnosed, it is typically at an advanced stage. Therefore, it is essential to implement effective measures as early as possible to distinguish the characteristics of liver tumours and lymph nodes, and to develop a comprehensive and effective treatment plan. This approach is crucial for improving patient prognosis and recovery (3).

In clinical practice, lymph node (LN) size, as assessed by computed tomography (CT) or magnetic resonance imaging (MRI), serves as the primary criterion for identifying pathological lymph nodes. A lymph node with a short-axis diameter exceeding 1 cm is typically classified as pathological. However, the threshold for benign lymph nodes varies depending on their anatomical location and the type of tumour involved (4).

Positron emission tomography (PET)/CT using [^18^F]2-deoxy-2-fluoro-D-glucose ([^18^F]FDG) combines functional metabolic imaging with anatomical structural imaging and has been extensively employed for the assessment, staging, and treatment monitoring of malignant tumours (5, 6). ^18^F-FDG PET-CT imaging can provide more reliable information, particularly in diagnosing whether lymph nodes are metastatic, as it primarily reflects the metabolic uptake of the lymph nodes and is less influenced by their size. PET scans can aid in identifying metastatic lymph nodes in patients, facilitating the biopsy and excision of suspicious lymph nodes located within dense lymphatic tissue (7). However, there is currently no standardized criterion for assessing lymph node metastasis based on the imaging characteristics of ^18^F-FDG PET-CT. It is generally accepted that when the SUVmax of lymph nodes is ≥ 2.6, tumour metastasis is likely present in those lymph nodes, although its correlation with pathological results is moderate to poor (8, 9). Nevertheless, the SUVmax values indicative of lymph node metastasis can vary depending on the tumour type (24, 25).

This study aims to compare and analyze the diagnostic value of ^18^F-FDG PET-CT imaging and enhanced abdominal CT scans in the preoperative detection of lymph node metastasis in liver cancer. Using Receiver Operating Characteristic (ROC) curve analysis, the study will determine the optimal diagnostic thresholds for lymph node SUVmax and the short-axis diameter of lymph nodes, as well as evaluate their diagnostic efficacy. The objective is to improve the accuracy of liver cancer staging, assist clinicians in developing personalized treatment plans, and ultimately enhance patient prognosis.

In comparison with the study conducted by Kawaoka (26) et al., which similarly concluded that PET-CT demonstrates superior sensitivity and specificity for detecting lymph node metastasis relative to multi-detector helical computed tomography, our investigation presents several notable advancements. Firstly, we employed integrated ¹^8^F-FDG PET-CT, enabling the concurrent evaluation of both metabolic activity and anatomical structure. Secondly, rather than applying a uniform threshold, we utilized ROC analysis to establish optimal, data-driven diagnostic cut-offs for SUVmax and the short-axis diameter of lymph nodes, tailored specifically to our study cohort. Most critically, we developed and validated a comprehensive diagnostic model that synthesizes these parameters. This multimodal strategy seeks to address the limitations inherent in relying solely on either size or metabolic criteria, thereby offering a more precise and clinically relevant tool for preoperative lymph node staging in liver cancer.

## 2 Material and methods

### 2.1 Data collection process and data items

A retrospective analysis of imaging data was conducted on 60 patients with newly diagnosed liver cancer who were hospitalized at Yueyang Hospital of Hunan Normal University between January 2019 and February 2025. All patients underwent concurrent ^18^F-FDG PET-CT and contrast-enhanced CT scans. Patients were rigorously selected according to specific inclusion and exclusion criteria, resulting in a cohort of 37 males and 23 females, aged 45 to 83 years, with a mean age of 64.73 ± 9.76 years. Tumour diameters ranged from 1.3 to 8.5 cm, and the body mass index (BMI) varied from 17.3 to 29.1. Patients were classified by pathological type into hepatocellular carcinoma, cholangiocarcinoma, and mixed cell carcinoma, and by tumour differentiation into poorly differentiated, moderately poorly differentiated, moderately differentiated, and well-differentiated categories. Using pathological results as the gold standard, the patients were further divided into a lymph node metastasis group (n=26) and a non-lymph node metastasis group (n=34). It was determined that the aforementioned clinical pathological indicators were not significantly associated with lymph node metastasis (P > 0.05) (**Table 1**). This study adhered to the ethical principles outlined in the Declaration of Helsinki and received approval from the Ethics Committee of Yueyang Hospital Affiliated with Hunan Normal University. Informed consent was waived as there were no potential risks to the patients included.

**Table 1:** General information of positive and negative groups in lymph node metastasis of liver cancer patients.

Group	Case no.	Positive lymph node metastasis(n=26)	Negative lymph node metastasis(n=34)	*X* ^2^	*P* Value
Age (year)				0.392	0.531
≤45 years	25	12	13		
>45 years	35	14	21		
Sex				1.448	0.229
Male	37	14	23		
Female	23	12	11		
BMI index				0.832	0.660
<18.5	18	7	11		
18.5-24	27	11	16		
≥24	15	8	7		
tumour size				0.274	0.601
<5cm	37	17	20		
≥5cm	23	9	14		
Pathological types				1.482	0.483
Hepatocellular carcinoma	24	10	14		
Cholangiocarcinoma	30	12	18		
Mixed-type liver cancer	6	4	2		
Degree of differentiation				0.639	0.887
Low differentiation	14	7	7		
Low-medium differentiation	11	4	7		
Medium differentiation	22	10	12		
High differentiation	13	5	8		

### 2.2 Inclusion criteria

Inclusion Criteria: (1) All patients underwent radical surgery for liver cancer, including regional lymph node dissection, with the time interval between the ^18^F-FDG PET-CT and CT scans and the surgery not exceeding one week; (2) All patients had liver cancer confirmed by histopathological examination; (3) No adjuvant treatments, such as radiotherapy or chemotherapy, were administered prior to surgery; (4) The ^18^F-FDG PET-CT demonstrated measurable positive lesions; (5) Complete clinical and pathological data were available. A total of 60 patients were included in the study.

Exclusion Criteria: (1) Patients with a history of other malignant tumours or distant metastases; (2) Patients who received radiotherapy, chemotherapy, or other forms of immunotherapy prior to surgery. 13 patients were excluded from the study.

### 2.3 Imaging examination instruments and methods

#### 2.3.1 PET-CT examination

The uMI510 PET-CT scanner from United Imaging, based in Shanghai, China, was utilized with the imaging agent ^18^F-FDG (radiochemical purity > 95%). Prior to the examination, patients were instructed to fast for more than six hours, and their height and weight were recorded. Fasting blood glucose levels were confirmed to be within the normal range (< 11.1 mmol/L). Following the intravenous injection of ^18^F-FDG at a dose of 3.70-5.55 MBq/kg, patients were asked to rest quietly for 60 minutes. They were then instructed to empty their bladders and remove any metallic objects from their bodies. CT and PET images were acquired sequentially, covering the area from the upper femur to the base of the skull, with a collection time of three minutes per bed position. After data acquisition, CT data were used to perform attenuation correction on the PET images. Following iterative reconstruction, horizontal, coronal, and sagittal PET, CT, and PET-CT fusion images were generated.

#### 2.3.2 Multilayer spiral enhanced CT examination

A Siemens 64-slice multilayer spiral CT scanner from Germany was used for the examination. Prior to the procedure, patients were instructed to fast for more than six hours and to cleanse their intestines. During the examination, patients were positioned in a supine manner and instructed to hold their breath while scanning from the diaphragm to the lower edge of the pubic symphysis. Following the completion of the initial scan, a dual-phase enhanced scan was performed. A dual-chamber high-pressure injector was used to administer 70-80 mL of iodinated contrast agent via the antecubital vein at a flow rate of 3.5 mL/s for dynamic enhancement scanning. After the scanning process was completed, the images underwent post-processing.

#### 2.3.3 Image analysis

**Figure 1:**
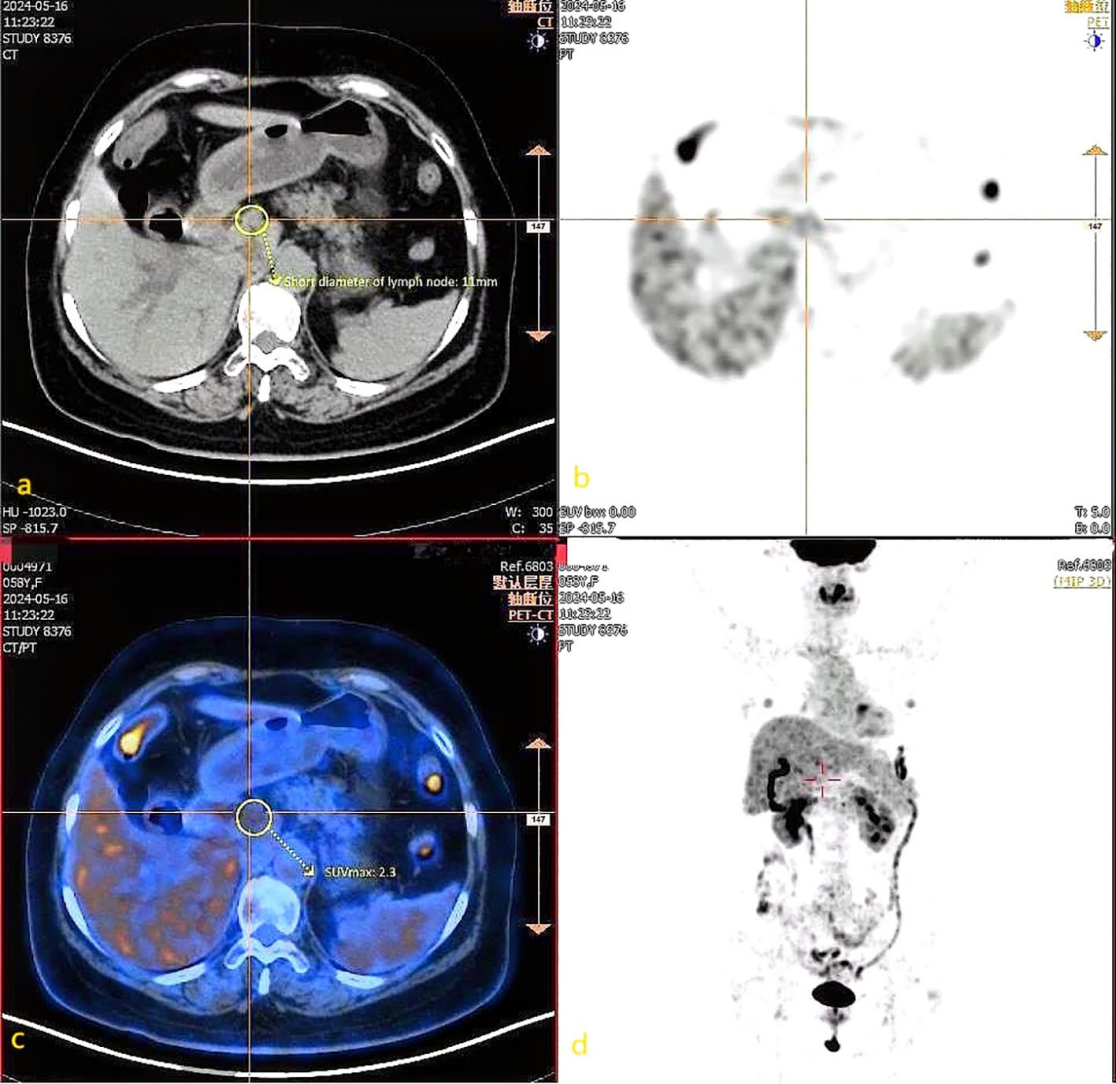
CT images and ^18^F-FDG PET-CT images of metastatic lymph nodes in liver cancer patients. **(a)** CT axial image shows a lymph node with a short-axis diameter of 11 mm. **(b)** PET images primarily reflect the metabolic activity of tissues, and the labeled areas may indicate regions with potentially abnormally increased metabolism. **(c)** The PET-CT fusion image shows the marked lymph node with an SUVmax of 2.3. **(d)** PET whole-body maximum intensity projection images can comprehensively display the metabolic activity of the entire body.

Two attending physicians with extensive clinical diagnostic experience independently interpreted the ^18^F-FDG PET-CT and CT images using a blinded method. In cases of disagreement between the observers, an independent senior nuclear medicine physician with 15 years of experience was consulted for final arbitration. The value of the two examination methods was assessed by comparing the lymph nodes detected and the corresponding parameters against on the pathological diagnosis results. **Figure 1** illustrates representative imaging findings of metastatic lymph nodes in a patient with liver cancer. This figure highlights the complementary roles of CT (morphological assessment) and PET (metabolic assessment) in evaluating lymph node involvement, emphasizing the clinical utility of integrated PET-CT imaging.

### 2.4 Observation indicators

This study compares the SUVmax, the short diameter of lymph nodes, and the diagnostic efficacy of ^18^F-FDG PET-CT versus CT in two groups: those with lymph node metastases and those without. Key metrics for evaluation include sensitivity, specificity, and accuracy. The SUV is the most commonly used parameter in ^18^F-FDG PET-CT imaging, with SUVmax representing the highest level of metabolic activity within tumour tissue. SUVmax is closely associated with the nature, grading, and staging of tumours; generally, a higher SUVmax indicates a greater degree of malignancy (10). Additionally, changes in the short diameter of lymph nodes can also suggest the presence of metastatic lymph nodes to some extent (11).

### 2.5 Statistical methods

Statistical analysis was conducted using SPSS version 26.0. Categorical data are presented as frequencies. For continuous data with a normal distribution, homogeneity of variance was assessed, while non-normally distributed continuous data are expressed as medians with interquartile ranges. Chi-square tests were used to compare categorical variables, while non-parametric tests were applied for comparisons involving non-normally distributed or ordinal data. Correlation analyses were conducted to examine the relationships between ^18^F-FDG PET-CT parameters and lymph node status, followed by multivariate logistic regression analysis to identify independent factors influencing lymph node metastasis. A combined diagnostic approach was employed to construct a predictive model ROC curve, and the diagnostic performance of this model was evaluated. The DeLong test was used to compare the areas under the ROC curves (AUCs). A p-value of less than 0.05 was considered statistically significant.

## 3 Results

### 3.1 Comparison of the diagnostic value of ^18^F-FDG PET-CT and CT for detecting lymph node metastasis in liver cancer

This study used pathological results as the gold standard to evaluate 60 patients diagnosed with liver cancer. The findings indicated that, among the patients with lymph node metastasis confirmed by pathology, there were 26 cases in total, of which 24 were consistent with the ^18^F-FDG PET-CT diagnosis. However, 2 cases were incorrectly diagnosed by ^18^F-FDG PET-CT as negative for lymph node metastasis. In the group without lymph node metastasis, as diagnosed by pathology, there were 34 cases, of which 30 were consistent with the ^18^F-FDG PET-CT diagnosis, while 4 cases were misdiagnosed by ^18^F-FDG PET-CT as positive for lymph node metastasis (**Table 2**). This resulted in a sensitivity of 92.31% (24/26), specificity of 88.24% (30/34), and accuracy of 90.00% (54/60) for the ^18^F-FDG PET-CT method. In contrast, for CT diagnosis, among the 26 cases in the positive group for pathological diagnosis of lymph node metastasis, 14 cases were accurately diagnosed, while 12 cases were incorrectly identified as negative for lymph node metastasis on enhanced CT. In the 34 cases of the negative group for pathological lymph node metastasis, 22 cases were correctly diagnosed, but 12 cases were mistakenly identified as positive for lymph node metastasis on enhanced CT (**Table 3**), yielding a sensitivity of 53.85% (14/26), specificity of 64.71% (22/34), and accuracy of 60.00% (36/60). The study concluded that the diagnostic efficacy of ^18^F–FDG PET-CT, in terms of sensitivity, specificity, and accuracy, was superior to that of CT, with statistically significant differences (*P* < 0.05) (**Table 4**).

**Table 2:** Comparison of ^18^F-FDG PET-CT and pathological diagnosis of lymph node metastasis.

^18^F–FDG PET-CT	Pathological examination		Total (N)
	Positive lymph node metastasis (N)	Negative lymph node metastasis (N)	
Lymph node metastasis	24	4	28
Non-lymph node metastasis	2	30	32
Total (N)	26	34	60

**Table 3:** Comparison of enhanced CT and pathological diagnosis of lymph node metastasis.

Enhanced CT	Pathological examination		Total (N)
	Positive lymph node metastasis (N)	Negative lymph node metastasis (N)	
Lymph node metastasis	14	12	26
Non-lymph node metastasis	12	22	34
Total (N)	26	34	60

**Table 4:** Comparison of the diagnostic efficacy of ^18^F-FDG PET-CT and CT for lymph node metastasis in liver cancer.

Detection method	Sensitivity (%)	Specificity (%)	Accuracy (%)
Enhanced CT	53.85 (14/26)	64.71 (22/34)	60.00 (36/60)
^18^F–FDG PET-CT	92.31 (24/26)	88.24 (30/34)	90.00 (54/60)
*X* ^2^	5.79	6.13	11.17
*P* Value	P<0.05	P<0.05	P<0.05

### 3.2 Correlation analysis of ^18^F-FDG PET-CT and enhanced CT parameters with lymph node status in liver cancer

This study conducted a univariate analysis of the parameters of lymph node short diameter and SUVmax. Following a normality assessment using the Shapiro-Wilk test, the results indicated that both parameters were normally distributed. Subsequently, a homogeneity of variance test (Levene’s test) was performed, revealing that the variance of the lymph node short diameter was homogeneous (P = 0.267). Therefore, an independent samples t-test was employed. In contrast, the variance of SUVmax was not homogeneous (P = 0.031), necessitating the use of Welch’ s corrected t-test. The results indicated that both the lymph node short diameter and SUVmax in the lymph node metastasis group were significantly higher than those in the non-lymph node metastasis group, with these differences being statistically significant (*P* < 0.05) (**Table 5**).

**Table 5:** Comparison of PET-CT and CT-related parameters with lymph node status.

Group	Non-lymph node metastasis(n=34)	Lymph node metastasis(n=26)	t	*P*
Lymph node short diameter	7.85 ± 2.32	9.92 ± 2.15	3.418	P<0.05
SUVmax	2.09 ± 0.51	2.86 ± 0.57	5.464	P<0.05

Logistic multiple regression analysis revealed that the Hosmer-Lemeshow test (HL test) yielded a *P*-value of 0.814, which exceeds the threshold of 0.05. This indicates no significant difference between the predicted values and the actual values, suggesting that the model demonstrates a good fit. The results of the logistic regression indicate that the short diameter of lymph nodes in CT parameters and the SUVmax in ^18^F-FDG PET-CT parameters are independent predictors of lymph node metastasis in liver cancer, and they are significantly positively correlated *(P* < 0.001) (**Table 6**).

**Table 6:** Multivariate logistic analysis of risk factors for regional lymph node metastasis in liver cancer.

Group	B	SE	OR(95%CI)	*P*
Lymph node short diameter	1.030	0.341	2.801 (1.437-5.460)	P<0.001
SUVmax	3.894	1.146	49.092 (5.199,463.547)	P<0.001

### 3.3 Efficacy analysis of optimal cut-off values for ^18^F-FDG PET-CT and CT-related parameters

**Figure 2:**
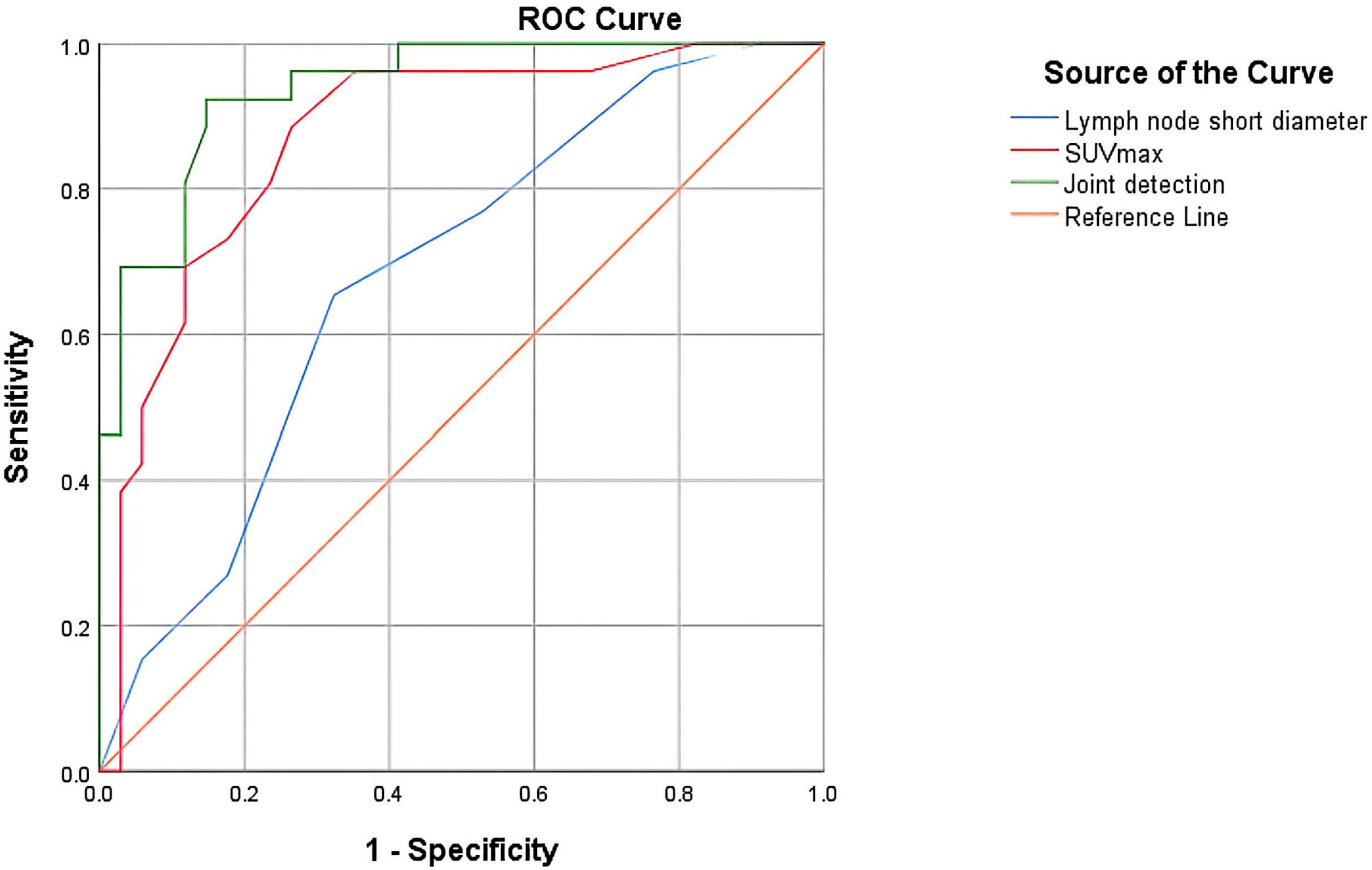
ROC curve evaluating the effectiveness of SUVmax, short diameter of lymph nodes, and their combination in diagnosing lymph node metastasis.

To determine the optimal thresholds for diagnosing lymph node metastasis in liver cancer using ^18^F-FDG PET-CT and enhanced CT imaging, this study plotted the ROC curves for lymph node short diameter, SUVmax, and their combined diagnostic approach (**Figure 2**). A larger area under the AUC indicates greater diagnostic accuracy, and the point closest to the upper left corner of the ROC curve—where the Youden index is maximized—was selected as the ideal diagnostic cutoff. When the threshold for SUVmax was set at 2.25, the Youden index reached its maximum, yielding an AUC value of 0.871. The sensitivity for diagnosing lymph node metastasis in liver cancer was 88.5%, specificity was 73.5%, and accuracy was 80.1%. From the ROC curve, the optimal threshold for lymph node short diameter was determined to be 8.5 mm, with an AUC value of 0.681, resulting in a sensitivity of 65.4%, specificity of 67.6%, and accuracy of 66.7% for predicting lymph node metastasis in liver cancer. To further enhance the diagnostic value of ^18^F-FDG PET-CT and CT, this study generated an ROC curve using the combined optimal thresholds of lymph node SUVmax and short diameter, resulting in an AUC value of 0.938. The sensitivity for the combined diagnosis of lymph node metastasis in liver cancer was 92.3%, specificity was 85.3%, and accuracy was 88.3% (**Table 7**).

**Table 7:** Predictive performance of short diameter of lymph nodes and optimal SUVmax threshold in lymph node metastasis of liver cancer.

Group	Threshold value	AUC	Sensitivity (%)	Specificity (%)	Accuracy (%)
Lymph node short diameter	8.5	0.681	65.4	67.6	66.7
SUVmax	2.25	0.871	88.5	73.5	80.1
Joint detection	–	0.938	92.3	85.3	88.3

Using the DeLong test to compare the AUCs of the three ROC curves pairwise, the results are as follows: the *Z* value between lymph node SUVmax and short diameter is 1.986, the *Z* value between lymph node SUVmax and combined detection is 2.043, and the *Z* value between lymph node short diameter and joint detection is 3.676. All differences are statistically significant *(P* < 0.05). This indicates that joint detection is more effective than single-parameter detection in diagnosing lymph node metastasis in liver cancer, which has clinical significance for the staging, treatment, and prognosis of the disease. Additionally, the diagnostic efficacy of lymph node SUVmax is superior to that of short diameter (**Table 8**).

**Table 8:** Comparison of the diagnostic value of the combined diagnosis of lymph node short diameter and SUVmax with that of individual parameters.

Group	*Z*	AUC difference	SE	95%CI	*P*
SUVmax-Lymph node short diameter	1.986	0.190	0.344	(0.002—0.378)	P<0.05
Joint detection-Suvmax	2.043	0.067	0.273	(0.003—0.113)	P<0.05
Joint detection-Lymph node short diameter	3.676	0.257	0.311	(0.120—0.395)	P<0.05

## 4 Discussion

Liver cancer poses a significant public health challenge worldwide. In developed countries, the increasing prevalence of obesity has made non-alcoholic fatty liver disease a major risk factor for the development of hepatocellular carcinoma (1). In several Asian countries, infections with hepatitis B virus (HBV) and hepatitis C virus (HCV) have emerged as important risk factors (12, 13). Primary liver cancer mainly comprises three types: hepatocellular carcinoma (HCC), intrahepatic cholangiocarcinoma (ICC), and combined hepatocellular-cholangiocarcinoma (cHCC-CCA). Of these, HCC accounts for 75% to 85% of cases, while ICC represents 10% to 15% (14). Primary liver cancer often has an insidious early onset, making early diagnosis challenging. Most patients are diagnosed at intermediate to advanced stages. Recurrence after treatment is common, and the survival rates remain low (15). Therefore, early detection, timely diagnosis, and prompt treatment are essential to improve survival outcomes for liver cancer patients. Imaging examinations, as the primary non-invasive method for early diagnosis, play a particularly important role (16).

Enhanced CT can clearly delineate the location of lesions, as well as the size, shape, and enhancement characteristics of lymph nodes, which differ significantly from those of normal tissue (17). With advances in imaging technology, the advent of PET has greatly improved our understanding of diseases at the genetic, molecular, metabolic, and functional levels. The principle underlying ^18^F-FDG PET-CT in detecting lymph node metastasis involves an energy-dependent process associated with the activation of inflammatory cells. Upon activation, these cells increase the expression of glucose transporters (GLUT) and hexokinase (HK) on their surfaces, thereby enhancing glucose uptake and utilisation. Similarly, ^18^F-2-fluoro-2-deoxy-D-glucose (^18^F-FDG), a radiolabelled glucose analogue, is absorbed at a higher rate by activated inflammatory cells and is retained within them. ^18^F-FDG PET-CT exploits this phenomenon to image inflammatory diseases. The liver, as the primary organ for glucose metabolism, contains specific glucose-6-phosphatase, which accelerates the metabolic processing of ^18^F-FDG ^(^18). Compared to normal liver tissue, liver cancer exhibits a reduced capacity to synthesize glucose-6-phosphatase. This deficiency results in a significant increase in intracellular levels of ^18^F-FDG, leading to a marked elevation in SUVmax (19). Furthermore, the parameters derived from ^18^F-FDG PET-CT may have predictive value for lymph node metastasis in patients with liver cancer (20, 21).

This study investigates the efficacy of ^18^F-FDG PET-CT and enhanced CT in assessing regional lymph node metastasis in patients with liver cancer, focusing on the imaging characteristics of regional lymph nodes. The results indicate that the sensitivity of ^18^F-FDG PET-CT for detecting regional lymph node metastasis in liver cancer is 92.31%, with a specificity of 88.24% and an accuracy of 90.00%. These values are significantly higher than those obtained from CT, with statistically significant differences (*P* < 0.05), demonstrating that the overall diagnostic value of ^18^F-FDG PET-CT surpasses that of CT. The sensitivity and specificity of CT in diagnosing regional lymph node metastasis in liver cancer are relatively low, primarily because the main criteria for diagnosing lymph node metastasis using CT are lymph node size and abnormal enhancement. Notably, among the 60 liver cancer patients studied, most of the metastatic lymph nodes were relatively small in size. Jiang et al. (22) found that small nodular or peripheral metastatic lymph nodes are typically smaller in size and are often challenging to diagnose. According to the RECIST criteria, metastatic lymph nodes must have a short-axis diameter of at least 15 millimeters on CT scans, while lymph nodes measuring less than 10 millimeters are considered normal. However, metastatic lymph nodes can be present in both larger and smaller lymph nodes, and not all large-diameter lymph nodes are necessarily metastatic. Therefore, relying solely on size as a criterion to assess lymph node status in liver cancer patients is inadequate. Furthermore, the enhancement pattern of lymph nodes does not have a definitive correlation with lymph node metastasis (17). Finally, the relatively low spatial resolution of CT images may contribute to these limitations, as they are easily influenced by partial volume effects. Furthermore, these techniques generally produce only cross-sectional images, which complicates the clear visualization of the morphology and internal characteristics of lymph nodes. This limitation also makes it challenging to identify small lesions or to differentiate between benign lymph nodes and potential metastatic lesions (23). Therefore, it is essential to utilize higher resolution and multi-plane reconstruction imaging techniques to achieve more accurate assessments of lymph nodes. The advent of ^18^F-FDG PET-CT has significantly addressed this challenge. ^18^F-FDG PET-CT imaging offers more reliable information, as its ability to diagnose metastatic lymph nodes is primarily based on the metabolic uptake capacity of the lymph nodes, which is less influenced by their size. PET scans can aid in identifying metastatic lymph nodes in patients, facilitating the biopsy and removal of suspicious lymph nodes situated among dense lymphatic tissue (7).

This study found that the two parameters, SUVmax and the short diameter of lymph nodes, were significantly higher in the lymph node metastasis group than in the non-lymph node metastasis group. Consequently, we conducted a logistic regression analysis and determined that the CT parameter of lymph node short diameter and the ^18^F-FDG PET-CT parameter of SUVmax are independent predictors of lymph node metastasis in liver cancer. Furthermore, as the lymph node SUVmax and short diameter increase, so does the likelihood of lymph node metastasis. Additionally, the study found that the AUC for lymph node SUVmax in diagnosing regional lymph node metastasis in liver cancer was 0.871, with sensitivity, specificity, and accuracy all superior to those obtained using lymph node short diameter alone. Based on the AUCs of lymph node short diameter and lymph node SUVmax (0.681 and 0.871, respectively), this study found that the combined diagnosis using the parameters of lymph node SUVmax and lymph node short diameter from ^18^F-FDG PET-CT has a high predictive value for regional lymph node metastasis in liver cancer. The AUC for predicting lymph node metastasis (0.938) is higher, with sensitivity (92.3%), specificity (85.3%), and accuracy (88.3%) all exceeding those of single parameter diagnoses. This improvement may be attributed to the combined approach enhancing morphological assessment while also indirectly reflecting the metabolic status within the lymph nodes through metabolic parameters, thereby improving the detection rate of metastatic lymph nodes to a certain extent. This has greater clinical guiding significance for the staging, treatment, and prognosis assessment of liver cancer.

However, this study has several limitations. Firstly, it is a single-center retrospective analysis, which may introduce bias into the results. Secondly, the sample size is relatively small, necessitating further large-scale stratified studies to explore and validate the effectiveness of combining lymph node SUVmax and short diameter in predicting regional lymph node metastasis in liver cancer using ^18^F-FDG PET-CT.

## 5 Conclusion

In summary, ^18^F-FDG PET-CT is an effective technique for the preoperative evaluation of regional lymph node metastasis in liver cancer, demonstrating diagnostic performance superior to that of enhanced abdominal CT scans. Utilising the optimal threshold method can further improve the diagnostic accuracy of ^18^F-FDG PET-CT for detecting regional lymph node metastasis in liver cancer. The proposed criteria for lymph node metastasis were an SUVmax greater than 2.25 or a short diameter exceeding 8.5 mm. This information can inform the development and optimisation of treatment plans for patients with liver cancer.

## Data Availability

The original contributions presented in the study are included in the article/supplementary material. Further inquiries can be directed to the corresponding author.
